# Advances and Challenges in Structural Studies of Bioactive Peptide-Anthracycline Conjugates: A Mass Spectrometric Insight

**DOI:** 10.3390/ijms26104896

**Published:** 2025-05-20

**Authors:** Eszter Fehérvári, Katalin Uray, Gitta Schlosser

**Affiliations:** 1 MTA-ELTE Lendület (Momentum) Ion Mobility Mass Spectrometry Research Group and Department of Analytical Chemistry, Institute of Chemistry, ELTE Eötvös Loránd University, Pázmány Péter sétány 1/A, H-1117 Budapest, Hungary; 2 Hevesy György PhD School of Chemistry, ELTE Eötvös Loránd University, Pázmány Péter sétány 1/A, H-1117 Budapest, Hungary; 3 HUN-REN–ELTE Research Group of Peptide Chemistry, Hungarian Academy of Sciences, Institute of Chemistry, ELTE Eötvös Loránd University, Pázmány Péter sétány 1/A, H-1117 Budapest, Hungary; katalin.uray@ttk.elte.hu

**Keywords:** anthracyclines, daunomycin, peptide–drug conjugates, mass spectrometry, electrospray ionization, MALDI, tandem mass spectrometry, fragmentation, LC-MS

## Abstract

Drug conjugates, in which chemotherapeutic or cytotoxic agents are coupled to targeting or delivering macromolecules like peptides or proteins via a linker, revolutionize cancer treatment. While protein-drug and antibody-drug conjugates have already secured a role in clinical oncology, peptide–drug conjugates (PDCs) are emerging as a promising alternative, offering enhanced efficacy and fewer side effects compared to the free drug molecules. Comprehensive chemical and biological investigation of PDCs is crucial during drug development and optimization, paving the way for the next generation of targeted therapies. Anthracycline-containing peptide conjugates have emerged as promising candidates in targeted cancer therapies due to their ability to deliver cytotoxic agents directly to tumor cells. However, their structural complexity poses significant analytical challenges, particularly in mass spectrometric characterization. Accurate identification and quantification of these conjugates are critical for assessing their stability, efficacy, and mechanism of action. This article explores the major difficulties encountered during mass spectrometry (MS) analysis of anthracycline-peptide conjugates, focusing on ionization issues, fragmentation behavior, and challenges of detection from biological matrix.

## 1. Introduction

Cancer remains one of the greatest public health challenges and is the second leading cause of death worldwide [1]. Depending on the tumor’s type and stage, treatment options typically include surgery, radiation therapy, or chemotherapy. In recent years, significant advancements in drug development have emerged; however, chemotherapy is still often accompanied by severe toxicity and side effects. These adverse reactions frequently limit the dosage or even the use of these drugs [2].

Despite breakthroughs such as immunotherapy, off-target cytotoxicity continues to pose a major obstacle in cancer treatment with small-molecule drugs. Enhancing tumor-targeting specificity is a promising strategy to minimize toxicity and improve therapeutic outcomes. One innovative approach involves the use of antibody-drug conjugates (ADCs)—chemotherapeutic agents linked to antibodies that can selectively target antigens overexpressed or mutated on cancer cells. While ADCs offer significant advantages over conventional small-molecule chemotherapies, they are not without challenges. Issues such as antibody aggregation, premature ADC dissociation, rapid renal clearance, poor tumor penetration, off-target toxicity, and potential immunogenicity of monoclonal antibodies still need to be addressed [3]. Overcoming these limitations is crucial for maximizing the therapeutic potential of ADCs and advancing cancer care.

Peptide-based cancer-targeting therapeutics use peptides that possess the unique ability of selectively binding to receptors that are overexpressed in various tumors, opening up a wealth of opportunities in pharmaceutical research. Their biocompatibility, ease of synthesis, and cost-effectiveness make them highly attractive, especially for large-scale production. Additionally, peptides offer remarkable chemical diversity, with their molecular weight and regioselectivity being precisely tunable. Importantly, peptides exhibit significantly lower immunogenicity compared to antibodies, further solidifying their potential as versatile and efficient tools in targeted cancer therapy. Several peptide–drug conjugates have progressed into clinical development, and a few have reached late-stage clinical trials or even market approval. A summary of selected PDCs in clinical development is shown in Table 1. In 2008, FDA approved the first peptide–drug conjugate, Lutathera (Lutetium-177 DOTA-Tyr3-Octreotate), which is a peptide–receptor radionuclide therapy agent, consisting of a somatostatin analog peptide linked to the radioactive isotope lutetium-177, via the chelator DOTA [4].

Thanks to their unique properties, peptide–drug conjugates (PDCs) are rapidly outpacing small-molecule drugs and antibody–drug conjugates in versatility and application. Beyond cancer therapy, PDCs are proving their potential in treating a wide range of diseases, including COVID-19, diabetes and rheumatoid arthritis [4]. Their utility extends even further, such as imaging, biosensing [5], and biocatalysis [6], making them a cornerstone in the future of therapeutic and diagnostic innovation.

## 2. Structural Complexity and Biological Effects of Anthracycline-Peptide Conjugates

**Figure 1:**
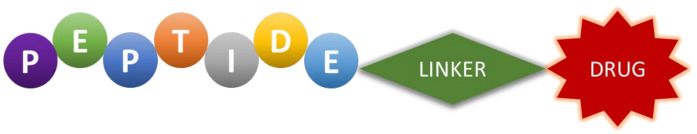
Schematic picture of peptide–drug conjugates.

Peptide–drug conjugates demonstrate superior efficiency compared to free drug molecules, offering a more targeted and effective approach to cancer therapy. A PDC is composed of three key components: a homing peptide, a linker, and a cytotoxic drug (Figure 1).

The homing peptide ensures precise tumor targeting, the cytotoxic drug delivers potent anticancer effects, and the linker serves as bridge connecting the drug to the peptide carrier and is responsible for the fine-tuned release of the drug (Table 2) [7]. Together, these structural elements create a powerful, synergistic system with improved specificity and reduced side effects.

Anthracyclines, such as daunorubicin, doxorubicin, epirubicin, and idarubicin, are among the most widely used antitumor agents, playing a pivotal role in the treatment of various cancers, particularly leukemias (e.g., acute myeloid leukemia, acute lymphoid leukemia) and neuroblastoma. A landmark in cancer therapy came in 1995 with the approval of Doxil, the first clinically applied doxorubicin-based drug, revolutionizing the treatment of Kaposi’s sarcoma [8].

**Figure 2:**
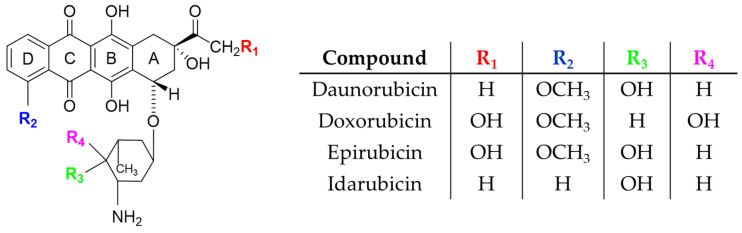
Structures of the main four anthracycline drugs.

The general structure of anthracyclines is distinctive and closely linked to their anticancer mechanisms of action. Their chemical composition is based on a tetracyclic A-B-C-D ring aromatic backbone derived from anthraquinone. The primary mechanism by which anthracyclines bind to Watson–Crick base pairs involves intercalation within the minor groove. The hydrophobic, aromatic D ring inserts itself perpendicularly between the base pairs, while the substituted A ring and the amino sugar align within the minor groove. The daunosamine sugar further stabilizes this interaction by forming hydrogen bonds in the minor groove. This intercalation disrupts DNA transcription and replication processes, thereby inhibiting the proliferation of cancer cells [9]. Anthracyclines also contain quinone and hydroquinone groups, which are redox-active. These groups generate reactive oxygen species (ROS), contributing to the destruction of cancer cells. However, this property is also associated with one of the major side effects of this drug class: cardiotoxicity. Additionally, various side chains are attached to the tetracyclic core and daunosamine, which determine the specific properties of individual derivatives and distinguish them from each other—examples are the doxorubicin and the daunorubicin. For instance, in the case of doxorubicin, the hydroxyl group at the 14th position (R_1_) plays a significant role (Figure 2) [10].

The mechanism of action of anthracyclines involves not only intercalation but also the inhibition of the enzyme topoisomerase II. Anthracyclines stabilize the DNA-topoisomerase II complex, preventing the re-ligation of DNA strands, which ultimately leads to cell death. The aromatic backbone and the amino sugar work together to ensure the drug’s strong binding to DNA and its anticancer activity [11]. This complex structure makes anthracyclines highly effective chemotherapeutic agents, but their use requires careful monitoring due to their associated side effects.

Anthracycline antibiotics, particularly doxorubicin and daunorubicin, are widely used anticancer agents. Doxorubicin is used to treat breast cancer, leukemias, and bladder cancer, while daunorubicin is mainly used in acute and chronic leukemias. While these compounds exhibit significant tumoricidal activity, their clinical application is often limited by toxicity and reduced efficacy against resistant tumor types.

Experiments demonstrated that the 2-pyrrolino modification of doxorubicin (e.g., 2-pyrrolino-doxorubicin) can increase its anticancer activity by 500–1000-fold in vitro. However, this modification rendered the compound excessively toxic, making it unsuitable for clinical use [12]. As an alternative approach, some studies proposed conjugating doxorubicin at the 14-OH position to achieve targeted therapeutic effects (e.g., with somatostatin or LH-RH analogs). However, these modifications reduced the broad-spectrum efficacy of the compound, as they were only effective against specific tumor types [13].

In contrast, the novel 2-pyrrolino-13-deoxyanthracyclines provide enhanced anticancer efficacy while maintaining broad-spectrum activity and exhibiting reduced toxicity. The most significant new compounds include: 2-pyrrolino-13-deoxydoxorubicin, 2-pyrrolino-13-deoxydaunorubicin, 2-pyrrolino-13-deoxyepirubicin, and 2-pyrrolino-13-deoxyidarubicin [12,13]. These newly developed compounds retain the mechanism of action of anthracyclines, specifically topoisomerase II inhibition and DNA intercalation, but structural modifications result in improved toxicity profiles and pharmacokinetic properties. Overall, the novel 2-pyrrolino-13-deoxyanthracycline derivatives represent a significant advancement in cancer therapy, as they provide enhanced efficacy with reduced toxicity and offer the potential for combination therapies to further optimize anticancer effects [12].

Another way to enhance the drug’s anticancer efficacy while reducing its systemic toxicity is its encapsulation in liposomes. Liposomes, as lipid-based vesicles, enable controlled drug release and targeted delivery, minimizing damage to healthy tissues. In contrast to its non-liposomal form, which exhibits significant toxicity; the liposomal formulation of 2-deamino-2-pyrrolino-daunorubicin demonstrated improved therapeutic potential, particularly against drug-resistant tumors, including those overexpressing P-glycoprotein. Liposomal formulation not only enhances the drug’s antitumor activity but also mitigates the adverse effects commonly associated with conventional chemotherapy, making it a promising candidate for cancer treatment [13].

**Figure 3:**
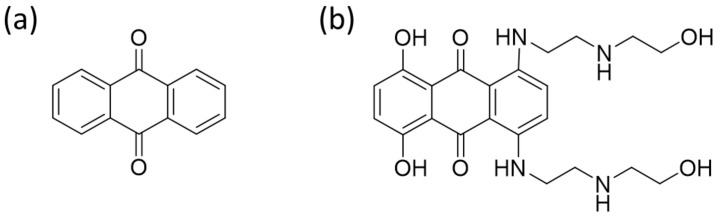
Chemical structure of the anthracenedione (**a**) and the mitoxantrone (**b**) molecules.

Anthracenediones (e.g., mitoxantrone and ametantrone) are structurally related to anthracyclines because both feature planar, aromatic ring systems that facilitate their intercalation into DNA, disrupting replication and transcription processes. Both drug classes are chemotherapeutic agents commonly used in oncology. Anthracenediones have a tricyclic aromatic backbone, which is less complex and lacks the sugar group (Figure 3). Due to their free radical production, anthracyclines are more likely to cause cardiotoxicity, especially in cumulative doses. Anthracenediones, such as mitoxantrone, exhibit reduced cardiotoxicity but may be associated with other toxicities, such as myelosuppression.

Multidrug resistance (MDR) presents a significant challenge in post-chemotherapy treatment, necessitating the development of innovative therapeutic strategies. A pH-responsive nanovesicle was recently developed, encapsulating mitoxantrone and chloroquine, enabling the co-delivery of both drugs. Chloroquine plays a crucial role in inhibiting mitoxantrone efflux and autophagy, thereby reversing mitoxantrone resistance. This novel formulation extended circulation time, facilitated synchronous dual-drug delivery, and enhanced the tumor targeting, offering a promising formulation for the treatment of mitoxantrone-resistant tumors [14].

In another study, a mitoxantrone-insulin conjugate was synthesized and characterized, in which the in vitro stability, in vivo biodistribution, tumor-targeting properties, and pharmacokinetics were studied in mice bearing H22 hepatocarcinoma. Since insulin receptors are highly expressed on the cytomembrane of certain tumor cells, the use of insulin as a vector was expected to reduce the severe side effects of the drug and it was hypothesized that the conjugate would selectively target solid tumors. In vivo studies on H22 hepatocarcinoma revealed that the conjugate exhibited good tumor targeting efficiency and showed significantly lower toxicity to other tissues compared to the free drug [15].

In the early 2000s, the development of peptide conjugates containing anthracycline-type drugs came to the forefront. Several early studies focused on the development of doxorubicin- and daunorubicin-based conjugates with peptides to enhance targeting specificity and reduce side effects [16,17,18,19,20]. Peptides can specifically bind to certain receptors found on the surface of cancer cells. In peptide–anthracycline conjugates, the peptide and the anthracycline are chemically linked, and the conjugate is directed to the desired target cells, which the peptide’s receptors recognize. This mechanism allows peptides to enhance the cellular uptake of drugs and reduce unwanted distribution throughout the body. Peptides for drug conjugation can be of various types, such as hormone peptides (like human calcitonin, GnRH-III), or short sequences with positive charges that aid in the passage of drugs through cell membranes (such as oligoarginines). When selecting peptides for conjugation, an important factor is the receptor specificity: the peptide should only bind to the targeted cells, thereby maximizing drug efficacy and minimizing side effects. One of the greatest advantages of anthracycline–peptide conjugates is that they target the drug specifically to cancer cells. The targeted delivery often results in fewer side effects compared to traditional chemotherapy treatments. It was demonstrated that this type of drug delivery is particularly beneficial in osteolytic metastases [21], hormone receptor-positive cancers [22], and heart-friendly chemotherapy approaches [23,24]. Examples are the GnRH-III peptide conjugates with doxorubicin, which enhance selectivity against hormone receptor-positive cancer types [23], while oligoarginine peptides facilitate faster cellular uptake of chemotherapeutic agents, thus increasing their effectiveness [25,26,27]. The targeted drug delivery using peptides holds great promise for a wide range of clinical applications, offering potentially groundbreaking results in cancer therapy in the future.

The linker in peptide–drug conjugates also plays a crucial role in determining the stability, specificity, and efficacy of the conjugate. The linker not only serves as a covalent bridge between the peptide and the drug but also influences the release kinetics and bioavailability of the therapeutic agent. Based on their properties and activation mechanisms, linkers in PDCs can be classified into several categories (Table 2). An ideal linker moiety should exhibit high stability in systemic circulation, specific activation at the target site, and biocompatibility to minimize toxicity and immune response. A carefully selected linker can significantly enhance the therapeutic index of PDCs by ensuring efficient drug delivery while reducing off-target effects. In conclusion, linker engineering plays a fundamental role in PDC development, directly influencing drug stability, releasing kinetics, and targeting efficiency. Advances in linker design continue to improve the precision and efficacy of peptide–drug conjugates, paving the way for more effective and safer therapeutic strategies.

## 3. Analytical Control of Anthracycline–Peptide Conjugates: From Concept to Applications

The analytical control of peptide–drug conjugates (PDCs) is not just a formality—it is the backbone of robust chemical synthesis and drug development. Techniques like liquid chromatography-mass spectrometry (LC-MS) or nuclear magnetic resonance spectroscopy (NMR) are indispensable for verifying the structure and purity of these complex molecules. LC-MS, especially when combined with high-resolution mass spectrometry (HRMS), provides unparalleled precision, confirming molecular mass, elemental composition, and successful conjugation between drug and peptide components. Furthermore, tandem mass spectrometry (MS/MS) or enzymatic digestion enables detailed characterization of the drug–peptide linkage, ensuring that the intended chemical structure has been achieved.

Despite remarkable advances in analytical techniques, a surprisingly large number of studies still fail to report fundamental data such as molecular mass, compound purity, and mass spectra [28,29,30]. This critique is substantiated by several publications where key analytical results are either omitted, insufficiently detailed, or even manipulated. For instance, mass spectra are cropped with a paper strip, possibly to hide unidentified ions in the manuscript published by Ryppa et al. [31] Kalimuthu et al. [28] reported the synthesis and analysis of gold nanoparticle-stabilized novel peptide–drug conjugates but failed to provide any numerical MS data or readable mass spectra [28]. Similarly, Geng et al. [29] described a disulfide-linked PDC and provided detailed LC-MS/MS instrumentation parameters and quantification data, including calculated and measured molecular weights. However, no actual MS spectra were published, limiting the ability of readers to verify structural identity and fragmentation behavior [29]. The absence of these critical analytical data compromises reproducibility and limits confidence in the structural assignment. Encouragingly, recent publications reflect a positive shift towards greater transparency and analytical rigor. For example, the studies by Szabó et al. [32] or Baranyai et al. [33] provide detailed MS measurement parameters along with comprehensive monoisotopic mass data and ESI-MS spectra. These examples set a new benchmark for high-quality reporting in peptide–drug conjugate research and should be regarded as best practices to follow.

This review dives into the challenges and key considerations for the mass spectrometric analysis of anthracycline–peptide conjugates. It highlights best practices for achieving reliable results and offers a practical guide to interpreting experimental data with confidence. The time to tighten analytical standards is now—scientific progress demands nothing less.

## 4. Mass Spectrometry of Anthracycline–Peptide Conjugates

The hybrid nature of anthracycline–peptide conjugates combines hydrophobic and hydrophilic parts of very different structures, which can complicate mass spectrometric analysis. The anthracycline structure and the peptide backbone behave differently during ionization in the ion sources, leading to inconsistent ion yields and mass spectra. Furthermore, the presence of charged functional groups on the peptides and on the anthracycline moiety introduces competing ionization pathways. This is particularly problematic in electrospray ionization (ESI), where ion suppression or enhancement can occur depending on the surrounding matrix and the physicochemical properties of the analyte.

Mass spectrometry enables the detection of low concentrations of PDCs in biological matrices, overcoming the challenges posed by sample complexity. Two primary ionization techniques, Electrospray Ionization (ESI), and Matrix-Assisted Laser Desorption/Ionization (MALDI), are employed in combination with various MS platforms for this purpose. ESI-MS is the most commonly used technique for detecting PDCs in liquid samples. It provides high sensitivity and allows the analysis of intact conjugates as well as their metabolites. Coupling ESI-MS with liquid chromatography (LC-MS) enables the separation of the conjugate from endogenous biomolecules, such as proteins, lipids, or other metabolites, improving signal-to-noise ratios and quantification accuracy. MALDI-MS is a complementary technique that facilitates the rapid screening of biological samples. Although less commonly used for quantitative analysis, it is well-suited for analytical characterization of PDCs. Mass spectrometry, especially when coupled with liquid chromatography, is an indispensable tool for the detection and structural studies of anthracycline-containing peptide conjugates. Its ability to overcome the challenges of complex biological matrices while providing high sensitivity and specificity makes it ideal for pharmacokinetic studies, biomarker research, and drug development. As MS technologies continue to advance, they will further enhance our understanding of the behavior and efficacy of these conjugates in biological systems.

### 4.1. Analysis of Anthracycline-Peptide Conjugates Using Electrospray Ionization

Electrospray Ionization Mass Spectrometry (ESI-MS) is a powerful analytical technique for the characterization of these conjugates, providing insights into their molecular structure, binding properties, and stability. ESI-MS is particularly suited for the analysis of anthracycline-containing peptide conjugates due to its ability to ionize large, non-volatile, and thermally labile biomolecules without significant fragmentation. This “soft ionization” method facilitates the accurate determination of molecular masses, which is essential for confirming the successful conjugation of the anthracycline moiety to the peptide carrier. Moreover, ESI-MS enables the analysis of multi-charged ions, which extends the mass range of the instruments and enhances the analysis of large molecules.

When coupled with high-resolution mass spectrometry (HRMS), ESI-MS provides precise mass measurements, allowing the identification of specific structural features, including attachment sites, peptide sequence verification, and the presence of any post-synthetic modifications or degradation. Tandem mass spectrometry (ESI-MS/MS) further enhances this capability by enabling detailed structural elucidation of both the peptide backbone and the anthracycline–peptide linkage. Tandem mass spectrometry is achieved through fragmentation techniques such as collision-induced dissociation (CID) or higher-energy collisional dissociation (HCD). In addition to structural characterization, ESI-MS is a valuable tool for studying the solution-phase behavior of PDCs. It can provide information on their non-covalent interactions with biomolecular targets, such as DNA or proteins, offering insights into their mechanism of action. The ability of ESI-MS to interface with liquid chromatography (LC) systems also allows for the separation and analysis of complex mixtures, enabling the detection of impurities, degradation products, or multiple conjugation products in a single run.

**Figure 4:**
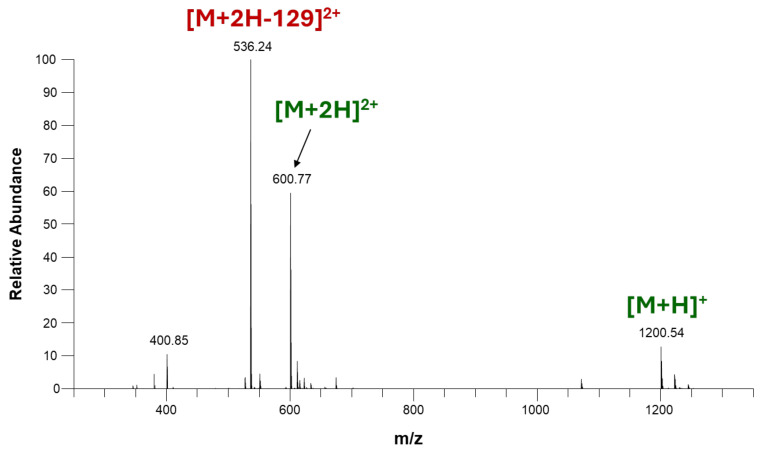
ESI spectrum of a daunomycin-peptide conjugate, Dau = Aoa-SKAAKN-OH. M (monoisotopic, calculated): 1199.53. Red label indicating the loss of a 129 Da unit from the doubly protonated molecule represents the loss of the daunosamine sugar in the ion source.

Despite its numerous advantages, ESI-MS faces challenges, including ion suppression effects caused by complex biological matrices or sample impurities. These can be mitigated through careful sample preparation and the use of appropriate solvents, additives, or chromatographic separation prior to ionization. Optimizing instrument parameters, such as spray voltage, flow rate, and desolvation temperature, is also critical for obtaining high-quality data. ESI-MS is a soft ionization technique optimal to analyze biomolecules, because it allows the ionization of peptides and proteins from solution in their intact forms. However, the major challenge during the mass spectrometric analysis of anthracycline–peptide conjugates is fragmentation within the ion source, which can significantly complicate the interpretation of the resulting spectra [34]. A typical ESI-MS spectrum of a daunomycin-peptide conjugate is shown on Figure 4, as a characteristic example for *in-source* fragmentation during ESI-MS.

Figure 4 shows the ESI spectrum of a Dau = Aoa-SKAAKN-OH conjugate, in which daunomycin (Dau) is conjugated to the targeting peptide (SKAAKN) by an aminooxyacetyl moiety (=Aoa) [35]. Under the commonly used ESI-MS conditions (water–acetonitrile solvent mixture with 0.1% V/V formic acid), the most intensive ion detected for this daunomycin–peptide conjugate corresponds to a fragment with sugar loss, instead of the intact protonated molecule. Singly and doubly protonated molecules are detected with lower intensities only. This unusually intensive fragmentation often occurs due to the inherent instability of the conjugates under the conditions present in the ESI ion source. When weak chemical bonds in the peptide–drug conjugate break apart, the mass spectrum becomes cluttered with fragment ions, making it difficult to identify and confirm the intact molecule and to interpret the origin of peaks other than the intact protonated ions. This presents a twofold problem. First, excessive fragmentation can obscure the detection of the intact molecule, which is essential for confirming the molecular mass and composition of the PDC. Second, it increases the risk of misinterpretation, as researchers might erroneously attribute fragment peaks to impurities or side products rather than *in-source* decomposition products of the conjugate. This can lead to inaccurate conclusions about the purity and structural integrity of the compound. It is important to note that while *in-source* fragmentation can also be observed for free anthracycline drugs, it is not significant under appropriate experimental conditions [34].

Fragmentation in the ion source is primarily driven by (a.) thermal and collisional stress: collision with gas phase solvent molecules and heat in the ionization chamber can destabilize fragile linkages, especially ester, amide, or other labile bonds in the conjugate; (b.) chemical instability: anthracycline–peptide conjugates inherently possess a weak glycosidic bond prone to cleavage under ionization conditions or in solution during synthesis; and (c.) ionization method: techniques like electrospray ionization (ESI) and atmospheric pressure chemical ionization (APCI) can exert different levels of energy on the molecule, with improper tuning exacerbating fragmentation. To reduce fragmentation and improve the reliability of mass spectrometric analysis, several approaches can be implemented. (1.) Optimize instrument parameters: lowering the source temperature and reducing ionization voltage can significantly decrease the energy imparted to the molecule, preserving the intact molecule [36]. (2.) Use even softer ionization techniques: soft ionization methods like matrix-assisted laser desorption/ionization (MALDI) can often produce better results for labile conjugates, as they may impart less energy compared to traditional electrospray techniques [37,38,39]. (3.) Use of non-acidic solvents to reduce the charge state of multiply protonated ions: high protonation levels have been observed to induce increased *in-source* fragmentation [34,40]. Replacing acidic solvents with neutral buffers significantly improves the ratio of intact molecules in the mass spectra. The authors recommend the use of 50 mM ammonium acetate (pH 6.7) solution mixed with acetonitrile (1:1, V/V) as a sample solvent when analyzing anthracycline–peptide conjugates by direct infusion or flow injection experiments in ESI-MS. Combining MS with orthogonal techniques like NMR can help validate results and circumvent issues related to in-source fragmentation. By implementing these strategies, researchers can achieve cleaner mass spectra, more accurate structural information, and ultimately more reliable data on anthracycline–peptide conjugates. Strengthening analytical rigor in this way is crucial for driving progress in PDC development and ensuring the reproducibility and reliability of scientific findings.

The selection of appropriate ESI-MS instrumentation is also crucial. While simple, low-resolution instruments (such as ion traps and quadrupoles) are well-suited for characterizing purified compounds, high-resolution systems—such as quadrupole time-of-flight (Q-TOF) or Orbitrap analyzers—may be required for the analysis of large molecular weight compounds or complex biological samples.

In conclusion, ESI-MS is a highly versatile and sensitive technique for the comprehensive analysis of anthracycline-containing peptide conjugates. Its ability to provide detailed molecular and structural information makes it an indispensable tool in the development and optimization of peptide-based drug delivery systems. Employing optimized ESI conditions, such as tuning the solvent composition and pH, can improve ionization efficiency and decrease the degradation in the ion source. Alternative ionization techniques like matrix-assisted laser desorption/ionization may overcome some ESI limitations, but they also require careful selection of experimental conditions.

### 4.2. Analysis of Anthracycline–Peptide Conjugates Using MALDI Methodology

One of the most promising analytical techniques for the routine analytical screening of PDCs is Matrix-Assisted Laser Desorption/Ionization mass spectrometry. MALDI-MS offers several advantages in the analysis of these conjugates, including high sensitivity, rapid analysis, minimal sample amounts and simple sample preparation. It enables fast molecular mass determination of the peptide conjugates and facilitates the identification of structural modifications or degradation products. The choice of the matrix is critical in optimizing spectrum quality; matrices such as α-cyano-4-hydroxycinnamic acid (CHCA) or 2,5-dihydroxybenzoic acid (DHB) are commonly used for peptide and small molecule analysis. Despite its strengths, MALDI-MS analysis of daunomycin-containing peptide conjugates also presents challenges, including ion suppression effects and the potential for in-source fragmentation. These can be mitigated by optimizing the sample preparation protocol, matrix selection, and laser intensity [41].

As it was demonstrated by Borbély et al. [41], the analysis of daunomycin conjugates by MALDI-TOF/TOF tandem mass spectrometry provides valuable insights into their structural features [41]. In particular, the use of MALDI-TOF/TOF with collision-induced dissociation (CID) activation was proved to be effective in characterizing the sequence of peptide backbones and determining conjugation sites. The dissociation patterns observed during these analyses are influenced by the position of the daunomycin moiety within the peptide sequence, revealing distinct fragmentation behaviors depending on whether the drug is conjugated to the *N*-terminus or a lysine side chain of the peptide.

**Figure 5:**
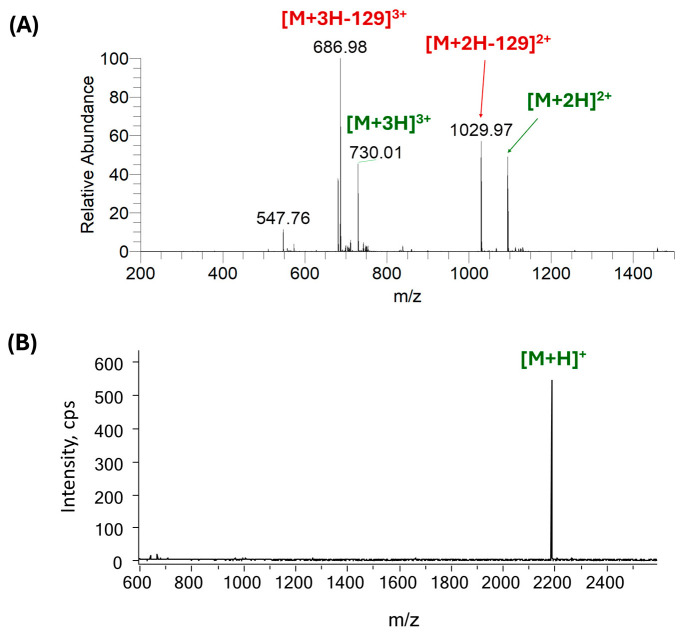
Comparison of mass spectra of Dau = Aoa-SYSNleEHFRWGKPV-NH_2_ conjugate measured using (**A**) electrospray ionization and (**B**) MALDI. Protonated ions labeled with green color are intact molecules, fragments with sugar-loss are depicted in red. Theoretical monoisotopic mass: 2186.0109; measured monoisotopic mass: 2186.0070 (ESI-orbitrap) and 2185.7 (MALDI-TOF).

To demonstrate the difference between the ESI-MS and the MALDI-MS technique, the spectra of a daunomycin–peptide conjugate are shown on Figure 5. In this construct, daunomycin was conjugated to an α-MSH–peptide analog (SYSNleEHFRWGKPV) using a non-cleavable oxime linkage (Aoa) to target melanocortin 1 receptor in malignant melanoma [32]. The conjugate was analyzed by ESI-MS (upper spectrum) and MALDI-MS (lower spectrum) under the commonly used experimental conditions. While the loss of the daunosamine sugar was dominant from the multiply protonated molecules under ESI conditions, producing artifact fragment peaks, the MALDI-MS verifies that the conjugate is indeed a single compound. In this case, only the intact, singly protonated molecule was detected in MALDI-MS, facilitating the verification of product quality (Figure 5).

Overall, MALDI-MS, when combined with optimized experimental parameters, is a highly effective approach for the structural characterization of anthracycline–peptide conjugates. MALDI-MS can also be used to assign conjugation sites and differentiate between conjugate isomers, which makes the technique a valuable tool for the characterization of PDCs.

### 4.3. Fragmentation Behavior and Tandem Mass Spectrometric Analysis

Structural studies of PDCs, i.e., the sequencing of peptide backbones and the verification of anthracycline attachment sites is performed by tandem mass spectrometry (MS/MS). This is crucial for confirming the conjugation chemistry and ensuring that the drug is appropriately linked to the peptide carrier. However, anthracycline-containing conjugates exhibit unusual fragmentation patterns due to the presence of various chemical bonds with highly different lability. As mentioned above, the spontaneous in-source fragmentation of anthracycline containing conjugates during electrospray ionization is usually significant, which influences the quality of the mass spectrometric results negatively.

The fragmentation of anthracyclines, such as daunomycin, exhibits several distinct features primarily related to the loss of sugar or daunomycin moieties, as well as complex fragmentation patterns involving the drug itself. Fragmentation pathways commonly observed for daunomycin conjugates are dominated by the neutral loss of the sugar and the daunomycin molecules, while peptide-related fragments often appear with low intensity.

One significant factor influencing anthracycline fragmentation is the chemical modification introduced by conjugation with peptides. This conjugation increases both the molecular weight and the gas-phase basicity of the conjugates, which in turn impacts the fragmentation efficiency. Higher energies and higher charge states are required to facilitate proton mobilization and promote charge-directed backbone cleavages compared to unmodified peptides in CID or HCD experiments. HCD is an effective fragmentation technique in orbitrap instruments, in which peptide ions are collided with an inert gas, transferring energy to them, which facilitates the fragmentation of the peptides along the amide bonds, resulting in the formation of *b*- and *y*-type ions. However, it was observed that HCD fragmentation of daunomycin–peptide conjugates predominantly resulted in the loss of the daunomycin sugar moiety only, and did not provide adequate sequential fragments, which ultimately limited the amount of structural information [42]. While HCD was capable of effectively fragmenting the unconjugated peptide, it did not provide sufficient data for the structural characterization of the conjugates. It was observed that the optimal selection of charge state and energy was crucial for peptide backbone fragmentation to improve the sequential coverage of the conjugates. Overall, while the use of the HCD technique can be successful for the fragmentation of peptide conjugates, the sequential information of the peptide is not always available. Selection of ions with higher charge states and fragmentation with greater collision energy are recommended for a more effective peptide backbone fragmentation.

In addition, anthracyclines in PDCs exhibit an “electron predator” effect, particularly relevant in electron-transfer dissociation (ETD) and electron-transfer/higher energy collisional dissociation (EThcD). ETD is an effective fragmentation method for identifying long and highly charged peptides generating *c*- and *z*-type fragment ions. It preserves post-translational modifications and other chemical modifications and provides more complete sequence coverage for modified peptides compared to CID and HCD. However, in many experiments, the inhibition of ETD fragmentation was observed likely due to the so-called “electron predator” effect. In this case, the spectra mainly contain charge-reduced precursor ions and sugar-lost fragments, while the fragments necessary for complete sequence determination were absent. The EThcD method was also ineffective for the structural analysis of daunomycin conjugates, as the collisional dissociation following the electron transfer did not result in sufficient backbone fragmentation. Consequently, neither ETD nor EThcD provided enough information to determine the peptide sequence or the drug conjugation site [41]. It can be concluded that anthracyclines, with their high electron affinity, inhibit radical-induced peptide backbone fragmentation by capturing electrons, preventing the cleavage of the peptide chain. This effect significantly hinders the sequencing of peptides conjugated with anthracyclines using the commonly used electron-based fragmentation techniques [41,42].

Compared to previous fragmentation techniques, MALDI-MS/MS provides significant advantages in the structural characterization of peptide–daunomycin conjugates. While HCD, ETD, and EThcD mainly resulted in daunomycin-related dissociation pathways with limited peptide backbone fragmentation, MALDI-MS/MS allowed for a more detailed analysis of peptide structures. MALDI-TOF/TOF enables various fragmentation techniques, including in-source decay (ISD) as a pseudo-MS/MS method, as well as laser-induced dissociation (LID) and high-energy collision-induced dissociation (high energy CID) as real MS/MS methods. The ISD technique exhibited low fragmentation efficiency for short daunomycin–peptide conjugates, resulting primarily in partial daunomycin degradation while providing limited structural data on the peptide backbone [41,43]. LID experiments revealed that fragmentation depends on the peptide length and the position of daunomycin in the conjugates, with characteristic fragment ions indicating specific cleavage sites. However, backbone-derived fragment ion intensities still remained low [41,44]. High-energy CID generated more informative product ion spectra in MALDI-MS, where certain peptide sequence-specific fragments had comparable or even greater intensity than daunomycin- or sugar-loss fragments. CID activation was particularly useful for identifying conjugation sites, as distinct fragmentation patterns enabled localization of modifications at lysine residues or the *N*-terminus [41,42]. Applying MALDI-MS/MS to complex peptide conjugates, such as daunomycin derivatives of Angiopep-2—a peptide ligand of low-density lipoprotein receptor-related protein-1—enabled differentiation between *N*-terminal and Lys side chain conjugation. The distinct fragmentation behavior of these conjugates facilitated unambiguous localization of daunomycin attachment, which was not achievable with other fragmentation methods [41].

Overall, MALDI-MS/MS outperformed previously used fragmentation techniques by generating more informative peptide backbone fragmentation and providing identification of conjugation sites. The presence of intense *a*- and *b*-ions, associated with peptide backbone cleavage near the conjugation site, can provide valuable information for localizing and verifying the conjugation site. This makes it a highly valuable tool in mass spectrometry-based structural studies, significantly enhancing the analytical characterization of peptide–daunomycin conjugates [41].

The sugar loss represents a critical fragmentation pathway in the case of anthracycline-based conjugates. The loss of the sugar moiety and the daunomycin molecule were observed across various mass spectrometry techniques, including HCD, ETD, EThcD, MALDI-ISD, MALDI-LID, and CID. Anthracyclines like daunomycin are tightly associated with a fragmentation pathway that primarily focuses on the removal of sugar groups, making this loss the most characteristic and dominant feature. Since the loss of the sugar and daunomycin molecules usually dominate the fragmentation spectra, insufficient structural information remains to precisely assign the conjugation site of the drug or identify the peptide sequence. The fragments resulting from sugar loss and daunomycin removal do not provide adequate or reliable sequence-specific data for peptide backbone analysis, which is a major challenge in the structural studies of anthracycline–peptide conjugates by tandem mass spectrometry.

Comparison of tandem mass spectrometry (MS/MS) results obtained using different instruments can be challenging due to variations in fragmentation methods and instrument configurations. MS spectra may also change over time as a result of fluctuations in instrument condition. To ensure long-term reproducibility, it is essential to optimize the collision energy to obtain high-quality MS/MS spectra. Even when using the same tune file (instrument settings), it is advisable to verify the instrument’s condition with a reference spectrum, as ion source contamination from prolonged use can affect instrument tuning and, consequently, alter the MS spectra [45,46,47]. It is advisable to optimize the collision energy (collision voltage) for MS/MS experiments, as the energy required for optimal fragmentation of modified peptides can differ significantly from that of unmodified peptides [48,49].

In summary, the fragmentation of anthracyclines is a complex process influenced by their chemical structure, the position of conjugation, and the fragmentation technique employed. The presence of electron-affine groups like daunomycin or doxorubicine introduces unique challenges in fragmentation analysis, while methods like MALDI-TOF/TOF offer promising solutions for studying large, complex peptide–drug conjugates. Despite the clear advantages of MALDI-TOF/TOF and CID/HCD techniques, the MS/MS spectra may still provide incomplete sequence coverage, making it challenging to fully characterize peptide–drug conjugates with a complex structure. Nonetheless, with careful selection and optimization of fragmentation methods, conditions and precursor charge states, these techniques can aid in identifying conjugation sites and facilitate structural characterization of anthracycline-based conjugates.

## 5. Liquid Chromatography Coupled with Mass Spectrometry (LC-MS) in the Analysis of Anthracycline-Containing Peptide Conjugates

The structural complexity of these conjugates necessitates robust analytical techniques for their detailed characterization, including the confirmation of conjugation, determination of purity, and analysis of stability. Liquid chromatography coupled with mass spectrometry (LC-MS) has emerged as a gold-standard method for studying such complex biomolecules, combining the separation power of liquid chromatography with the sensitivity and specificity of mass spectrometry. LC-MS is ideally suited for analyzing anthracycline-containing peptide conjugates due to its ability to resolve complex mixtures and provide high-quality mass spectral data. The LC component enables the separation of the conjugate from impurities, degradation products, and unconjugated starting materials, which is crucial for ensuring product purity and quality control. Reversed-phase LC (RP-LC) is most commonly employed due to its efficiency in separating hydrophobic and amphiphilic molecules. Employing C18 columns is standard for analyzing anthracycline–peptide conjugates, as it provides excellent separation based on hydrophobicity. Mobile phases typically consist of water and acetonitrile with additives like formic acid or trifluoroacetic acid (TFA) to enhance ionization and peak shape [40,50,51,52]. Hydrophilic interaction liquid chromatography can also be used for conjugates with high polarity, ensuring better separation of hydrophilic degradation products or impurities.

LC-MS plays a crucial role in confirming the successful conjugation of the anthracycline to the peptide carrier, while simultaneously identifying any unreacted starting materials or potential side products. This step is particularly critical in cases where extensive in-source fragmentation occurs, as it can complicate the interpretation of results. During the synthesis, the glycosidic bond in the anthracycline is susceptible to cleavage under acidic conditions, leading to the formation of a side product with diminished biological activity [33,53,54]. This issue is further compounded by the fact that the sugar-loss fragment generated during *in-source* fragmentation and the sugar-cleaved synthetic side product are structurally identical. As a result, careful separation is required to distinguish these species and verify the integrity and structure of the desired synthetic product. Advanced chromatographic techniques or complementary analytical methods may be necessary to achieve this level of resolution and ensure accurate characterization.

## 6. Challenges in Biological Sample Analysis

The detection of anthracycline–peptide conjugates in biological matrices presents several analytical challenges, including ion suppression from matrix components, low analyte concentrations, *in-source* fragmentation during MS analysis and potential degradation of the compounds during sample preparation. Biological samples, such as serum or cell lysates, introduce a complex matrix that interferes with MS analysis. Effective sample preparation is critical to minimize interference from complex biological matrices. Techniques such as protein precipitation, solid-phase extraction (SPE) and other desalting or purification methods are used to isolate the conjugate and remove unwanted matrix components, salts, buffers, or other contaminants to reduce ion suppression and improve the quality of mass spectrometric data. However, the authors recommend avoiding protein precipitation and ultrafiltration-based protein removal for anthracycline-containing samples, as these molecules noncovalently bind to proteins, which can cause a significant sample loss.

LC-MS enables the monitoring of conjugate stability under different conditions (e.g., pH, temperature), providing insights into degradation pathways and mechanisms [55]. LC-MS is applied in bioanalytical pharmacokinetic studies to measure the concentration of conjugates in biological matrices, providing data on their absorption, distribution, metabolism, and excretion. Degradation products, such as the free anthracycline or cleaved, anthracycline-containing peptide fragments, can be identified using LC-MS, providing insights into the metabolic degradation of the PDCs [46,56].

The degradation of bioactive anthracycline–peptide conjugates in lysosomal preparations is also a key area of study to better understand their intracellular stability and metabolic degradation. Lysosomal preparations, typically isolated from liver or other metabolically active tissues, are widely used. In such experiments, the rich content of proteolytic enzymes and acidic environment closely mimic the conditions inside cellular lysosomes. These models help simulate the enzymatic cleavage and degradation processes that conjugates may undergo in vivo [57,58]. Leurs et al. [58] developed GnRH-III based multifunctional drug delivery systems containing daunorubicin and methotrexate and investigated their in vitro stability in human serum and in the presence of rat liver lysosomal homogenate by LC-MS [58]. In this study, eight daunorubicin-bearing fragments of different lengths were observed, the smallest of them was *H*-K(Dau = Aoa)-*OH*—this metabolite can also effectively bind to DNA. However, the free daunorubicin could not be released from the lysine modified by aminooxyacetic acid.

In addition to lysosomal preparations, other methods are also employed to study the metabolism of peptide–drug conjugates, including cell-based assays and in vitro plasma stability tests [59,60]. These approaches provide complementary insights into the enzymatic stability and cleavage patterns of the conjugates under physiological conditions. Advanced analytical techniques, such as LC-MS and fluorescence-based assays, are routinely used to monitor the degradation products, ensuring a comprehensive understanding of the conjugate’s metabolic profile.

Ziaei et al. [61] conjugated doxorubicin to a triple-negative breast cancer specific, cell surface keratin 1 specific peptide carrier by the sulfosuccinimidyl 4-(N-maleimidomethyl)cyclohexane-1-carboxylate non-cleavable linker. The in vivo biodistribution of the conjugate in orthotopic breast cancer mouse model has been compared to that of doxorubicin using HPLC-MS and 7-fold higher tumor accumulation was found [61].

For pharmacokinetic and bioanalytical studies, tandem mass spectrometry (MS/MS) could also be employed for the selective and sensitive quantification of anthracycline-peptide conjugates; however, its successful use is still very limited in the literature. An important application field which necessitates MS/MS measurements is the highly complex biological samples, for example, liposome encapsulated formulations. Liposome encapsulation is an emerging research area to reduce the cytotoxicity of drugs [62,63]. PEGylated liposomes with targeting moieties are promising therapeutic agents, especially in highly metastatic experimental models. HPLC-MS/MS methodology was used in a mouse model by Vári et al. to characterize the cellular uptake, blood stability, and biodistribution of daunomycin encapsulated in peptide-targeted liposomes [64].

## 7. Outlook

Mass spectrometric analysis of anthracycline-containing peptide conjugates remains a challenging yet essential task for advancing their development as therapeutic agents. Addressing the ionization problems, unusual fragmentation patterns, and matrix effects through innovative analytical techniques and sample preparation strategies is crucial for accurate characterization. Further research into tailored MS approaches based on ESI-MS and MALDI-MS will be key to overcoming these challenges and unlocking the full potential of these conjugates in clinical applications. The integration of advanced high-resolution mass spectrometric techniques, along with optimized sample preparation methods, will significantly enhance the analytical characterization of anthracycline-peptide conjugates. These efforts will not only improve the accuracy and reliability of structural analysis but also pave the way for the development of more effective and targeted cancer therapies.

Peptide–drug conjugates are a key class of targeted therapeutics, with demonstrated advantages to enhance the physicochemical and biological properties of the highly toxic anthracyclines. Recent trends in anthracycline-containing peptide–drug conjugate research include novel, multifunctional targeting peptides, the use of novel cleavable linkers for controlled drug release and the application of advanced payloads. Characterization of these complex constructs, as well as the in vitro and in vivo bioanalytical measurements for drug development and clinical studies can only be performed by advanced mass spectrometric methodologies.

## Figures and Tables

**Table 1:** Examples of peptide–drug conjugates under clinical development.

Name	Target Disease	Clinical Phase	Drug/ Peptide Component
BT1718	Solid tumors	Phase I	DM1 toxin/ MMP-cleavable bicyclic peptide
PEN-221	Gastrointestinal neuroendocrine tumors	Phase II	DM1 toxin/ Somatostatin receptor- targeting peptide
Sudocetaxel zendusortide, TH1902	Solid tumors	Phase I	Docetaxel/ Sortilin-targeting peptide
Paclitaxel trevatide, ANG1005	Brain tumors (glioblastoma)	Phase II	Paclitaxel/ Angiopep-2
Zoptarelin doxorubicin, AEZS-108	Endometrial cancer	Phase III	Doxorubicin/ LHRH peptide

**Table 2:** The three main structural parts of peptide–drug conjugates.

Peptide	Linker	Drug
- Tumor-specific peptides	- Cleavable	- Cytotoxic agents
- Immunological targeting peptides - Enzyme-binding peptides	- Non-cleavable - Self-immolative - Spatially specific - Dual-functional	- Alkaloids and derivatives - Radiopharmaceuticals - Enzyme inhibitors - DNA-damaging agents

## Data Availability

Additional mass spectra are available on request.

## Abbreviations

The following abbreviations are used in this manuscript:

ADC	Antibody–drug conjugates
APCI	Atmospheric pressure chemical ionization
CHCA	α-cyano-4-hydroxycinnamic acid
CID	Collision-induced dissociation
DHB	2,5-dihydroxybenzoic acid
ESI	Electrospray ionization
ETD	Electron-transfer dissociation
EThcD	Electron-transfer/higher-energy collisional dissociation
FDA	Food and Drug Administration
HCD	Higher-energy collisional dissociation
HRMS	High-resolution mass spectrometry
ISD	In-source decay
LC	Liquid chromatography
LID	Laser-induced dissociation
MALDI	Matrix-assisted laser desorption/ionization
MDR	Multidrug resistance
MS	Mass spectrometry
MS/MS	Tandem mass spectrometry
NMR	Nuclear magnetic resonance spectroscopy
PDC	Peptide–drug conjugate
ROS	Reactive oxygen species
RP-LC	Reversed-phase liquid chromatography
SPE	Solid-phase extraction
TFA	Trifluoroacetic acid
TOF	Time of flight
